# Cervical ventral slot in rabbits (*Oryctolagus cuniculus*). Piezosurgery versus conventional technique

**DOI:** 10.1590/ACB360606

**Published:** 2021-07-09

**Authors:** Marcelo Roscamp, Alessandre Hataka, Frederico Carlini Zambon, Danyelle Rayssa Cintra Ferreira, Bruno Watanabe Minto, Luis Gustavo Gosuen Gonçalves Dias

**Affiliations:** 1 DVM, MSc. Department of Veterinary Medicine and Surgery – College of Agricultural Sciences and Veterinary Medicine – Universidade Estadual Paulista “Julio de Mesquita Filho” – Jaboticabal (SP), Brazil.; 2 DVM, PhD. Department of Veterinary Clinic – College of Veterinary Medicine and Animal Science – Universidade Estadual Paulista “Julio de Mesquita Filho” – Botucatu (SP), Brazil.; 3 DVM, Veterinarian. Anestesia Veterinária Especializada. Sao Paulo (SP), Brazil.; 4 DVM, MSc. Department of Veterinary Medicine and Surgery – College of Agricultural Sciences and Veterinary Medicine – Universidade Estadual Paulista “Julio de Mesquita Filho” – Jaboticabal (SP), Brazil.; 5 DVM, PhD. Department of Veterinary Medicine and Surgery – College of Agricultural Sciences and Veterinary Medicine – Universidade Estadual Paulista “Julio de Mesquita Filho” – Jaboticabal (SP), Brazil.; 6 DVM, PhD. Department of Veterinary Medicine and Surgery – College of Agricultural Sciences and Veterinary Medicine – Universidade Estadual Paulista “Julio de Mesquita Filho” – Jaboticabal (SP), Brazil

**Keywords:** Neurosurgery, Piezosurgery, Spinal Cord, Rabbits

## Abstract

**Purpose:**

To investigate the applicability of piezosurgery for cervical ventral slot (CVS), comparing it with the conventional technique of using high-speed burs for bone wear.

**Methods:**

Thirty rabbits (*Oryctolagus cuniculus*) were divided into two treatment groups (T1 and T2) corresponding to CVS between C3-C4. In T1, the surgery was performed with piezoelectric apparatus, and in T2 with high-speed burs. The evaluated parameters were: duration of each stage of surgery, temperature variations during CVS, visibility of the surgical field, intra and postoperative complications, and anesthetic monitoring. At 14, 28, and 56 postoperative days, five animals from each treatment group were submitted for histopathological study of the surgical site.

**Results:**

Compared with T2, T1 had more precise bone cut, and better visibility of the operative field, although it required longer total surgical time (p = 0.02) and triggered a greater number of intraoperative complications (p < 0.01), microscopic lesions in the spinal cord (p < 0.05), and transient neurological deficits in the postoperatively (p < 0.05).

**Conclusions:**

It is necessary to perform surgical planning and have several tips of the piezoelectric instrument available for the safe use of the piezoelectric device in neurosurgery.

## Introduction

Intervertebral disc disease (IVDD) is a common cause of neurological dysfunction^1^,^2^. Ventral slot is the surgical technique of choice of many neurosurgeons for the treatment of cervical IVDD, because it allows direct access to the herniated disc material, has shorter recovery period, and it is technically less demanding than the dorsal approach^3^.

High-speed burs are commonly used to promote vertebral bone wear. The perforation should be performed with caution, considering the changes in the coloration and texture of the bone tissue and identifying the neurovascular structures in order to avoid iatrogenic lesions^4^. However, excessive heat production and the possibility of injury to the adjacent soft tissues, even when the technique is performed cautiously, make these instruments disadvantageous^5^. Hence, piezoelectric technology was developed to address the need for greater safety and accuracy in bone surgeries in comparison to the traditional motorized instruments that were commonplace in surgeries until then^5^.

In piezosurgery, ostectomies are performed with ultrasonic instruments based on the piezoelectric effect. Micrometric vibrations are produced and transferred to the tip of the instrument, and the frequency of these vibrations ranges between 25 and 30 kHz, which can produce a mechanical cutting effect when applied with light pressure on the bone tissue. This particular frequency range ensures selective cutting of only the mineralized tissues, since soft tissues and neurovascular structures require frequencies higher than 50 kHz to be incised^5^-^10^.

Given the potential application of piezosurgery, especially in neurosurgery, and the scarcity of literature addressing the applicability of this technique, experimental studies are necessary. Therefore, the goal of this study was to investigate the applicability of piezoelectric surgery in the performance of cervical ventral slot (CVS), using rabbits (*Oryctolagus cuniculus*) as an experimental model for dogs and cats. For this purpose, the performance of CVS with piezoelectric apparatus and the one of high-speed burs (conventional technique) were compared. We hypothesized that piezoelectric surgery would have better intraoperative performance and superior postoperative results than the conventional technique.

## Methods

The present study was conducted with the consent of the corresponding Ethics Committee on the Use of Animals of the Universidade Estadual Paulista “Julio de Mesquita Filho”, Jaboticabal Campus (CEUA-FCAV-UNESP), under the number 5.403/16.

### Experimental design

Thirty white New Zealand breed of rabbits (*Oryctolagus cuniculus*) were used. The subjects were healthy adults, of the same age, with the average weight of 3.3 kg. Among them, 16 were female and 14 male.

The animals were divided into two treatment groups, and for each, one of the CVS techniques was performed. For T1, it was performed using a piezoelectric equipment, and for T2 the conventional technique was used, with a spherical drill-bit coupled to the high-rotation motor.

The Mastersonic medical device (VK Driller Equipamentos, São Paulo, SP, Brazil) was used in both treatments, and training for its operation was provided previously. Additionally, both techniques were performed by the same surgeon, alternately between the two treatment groups and in a surgical room at 25ºC.

Each treatment group (T1 and T2) was further divided into three groups: groups A and B at 14 postoperative days (POD), groups C and D at 28 POD, and groups E and F at 56 POD, based on the postoperative time elapsed until euthanasia. The groups A, C, and E belonged to T1 and B, D, and F to T2.

### Anesthesia and monitoring

The animals were submitted for preanesthetic medication with acepromazine (0.25 mg/kg, IM), midazolam (2 mg/kg, IM), and pethidine (3 mg/kg, IM), applied to the right pelvic limb. For anesthetic induction, 5% isoflurane was used with the aid of a face mask. Afterwards, all animals were orotracheally intubated and maintained in the anesthetic plane with isoflurane at the rate of 1 to 3%.

During the whole surgical procedure, as fluid therapy, the animals were maintained on continuous infusion of lactated Ringer’s solution at the rate of 10 mL/kg/h. All animals received tramadol hydrochloride (5 mg/kg, subcutaneous) and ketoprofen 10% (1 mg/kg, subcutaneous, every24 hours) and enrofloxacin 5% (10 mg/kg, subcutaneous, every 24 hours), both for five days.

Anesthesia monitoring was performed with a multiparametric monitor, for the following parameters: heart rate (bpm) and respiratory rate (mpm); systolic, diastolic, and mean arterial pressure (mmHg); oxygen saturation (%); concentration of carbon dioxide at the end of expiration (mmHg), and body temperature (ºC). These data, as well as the mean volume of isoflurane and fluid therapy required in the procedures, were recorded immediately before, during, and after CVS.

### Surgical procedure

After extensive trichotomy of the cervical region, the animals were placed in dorsal recumbency in the surgical gutter. Following rigorous antisepsis, the skin was incised, the subcutaneous tissue and the raphe of the muscles were adequately dissected, allowing the trachea, esophagus, and carotid sheath to be identified and retracted to the left side. The intervertebral space between the third (C3) and fourth (C4) cervical vertebrae was identified by digital palpation, and the muscles were elevated of the ventral face of C3 and C4.

CVS was performed including one-third the length and half the width of the vertebral body of the caudal portions of C3 and cranial of C4. Bone removal was performed in two ways, each corresponding to one type of technique. Therefore, T1 used piezosurgery, in which there was resection with Mastersonic piezoelectric apparatus containing a delicate chisel-type tip, pre-established surgical program with 70W of power (horizontal vibration of the tip), and 80W of modulation (vertical vibration of the tip), whereas for T2 the conventional technique was performed, in which the bone was removed with a carbide steel high-rotation spherical drill of 2 mm in diameter connected to the 1:1 straight hand piece, coupled to the surgical motor of the same Mastersonic device, with rotation of25,000 rpm and torque of 0.07 N.m. Both treatments received constant irrigation with sterile saline, at room temperature and at the rate of 30 mL/min, according to the technical indication of the equipment, and drainage was performed with a surgical aspirator.

In the T1 animals, slot ventral was performed *en bloc*, from cortical-to-cortical resection. In the T2 animals, following the principles of the conventional technique, when the internal cortex was reached, the bone removal was interrupted, and a Lucas curette no. 85 was used to remove it. After the conclusion of CVS, the same curette was used in both T1 and T2 animals to remove the remnant of the intervertebral disc (IVD), and any bleeding of the vertebral venous sinus was controlled with a lyophilized collagen hemostatic sponge. Finally, the closure of the soft tissues was performed as routine.

### Evaluation of surgical variables and data collection

During the surgical procedures in both treatments, the following variables were evaluated: surgical access time (from skin incision to exposure of the ventral face of C3 and C4), bone wear time, curettage time (IVD), and total time of the CVS procedure, and all of these measurements were performed with a stopwatch. The temperatures of the surgical room, the irrigation solution, and the CVS site were evaluated at three instances: immediately after the surgical access, during the technique, and at the end of the procedure. All the thermal measurements were performed at approximately 15 cm from the surgical site using infrared digital thermometer.

Additionally, the visibility of the surgical field was also assessed using the Likert-type scale, adapted from Farrell *et al*.^11^, in which score 1 means complete visibility, score 2 the presence of discrete bone bleeding and foam formation, score 3 the presence of moderate bone bleeding, controlled with a hemostatic sponge, and score 4 intense bone bleeding, making it impossible to conclude the procedure.

### Intraoperative complications

No intraoperative complication was recorded, including cardiac arrhythmias, cardiopulmonary arrest, and venous sinus hemorrhages.

### Postoperative neurological evaluation

Complete neurological examination was performed at 12, 24, 48, and 72 hours postoperatively.

### Histological evaluation

After the postoperative period scheduled for each group, the animals were submitted for euthanasia. The cervical columns were adequately dissected, prepared for histopathology, and were sent to be processed in the Research Laboratory of the Veterinary Pathology Service.

The histological sections were made at the ventral slot site. The reading of these slides was performed without the pathologist knowing which treatment or group was being analyzed. Thus, only at the end of the reading of all the slides, the results were attributed to their corresponding experimental group.

The changes suffered by the spinal cord were evaluated microscopically and staggered in:

- Absent (-): without signs of morphological lesion;
- Mild degree (+): discrete signs of Wallerian degeneration and spherocytes in up to 10% of the spinal cord;
- Moderate degree (++): Wallerian degeneration, spherocytes, malacia, and *Gitter cells* comprising 11 to 25% of the spinal cord;
- Severe grade (+++): Wallerian degeneration, spherocytes, malacia, and *Gitter cells* comprising 26 to 50% of the spinal cord.

### Statistical analysis

Statistical analysis was performed using STATISTICA 13.0 software (Tulsa, Oklahoma, United States). Except for the variable visibility of the surgical field and the histopathological analyzes that were subjected for Mann-Whitney test, all other variables and the consequent comparison between the treatments were analyzed using the Student’s t-test, with the significance level of 5%(p < 0.05) for the two tests performed. The variables related to the anesthetic monitoring were separated before, during, and after the CVS and submitted to the analysis of variance (ANOVA) and Student’s t-test. Pearson correlation was also performed between the variables, considering a strong correlation whenr > 0.75.

## Results

### Surgical procedure

Piezosurgery (T1) allowed for the execution of precise bone cuts, with regularity of the edges in a rectangular format with preservation of IVD. On the other hand, the use of the conventional technique with high-speed burs (T2) resulted in elliptical bone cut with irregularedges.

### Surgical variables

The intraoperative data are presented in Table 1. According to the Student’s t-test, only three variables showed significant differences (p < 0.05) between the treatment groups: curettage duration (p = 0.04), total procedure time (p = 0.02), and the difference between the initial and final temperatures at the CVS site during irrigation (p = 0.02). The parameters were higher in T1.

According to Mann-Whitney test, the visibility of the surgical field was also statistically different (p < 0.01) between the two treatment groups. The use of piezoelectric apparatus promoted better visibility than the conventional technique.

### Surgical times

In T1, the mean bone wear time was 8 min 43 s, and in T2, the mean bone wear time was reduced throughout the procedures. Although the difference was not statistically significant, bone wear time was higher in piezosurgery than in the conventional technique.

The curettage time was significantly higher in T1 than in T2. In the first procedures, it was very similar in both treatments. However, subsequently, there was a substantial increase in the curettage time in piezosurgery, which resulted in an increase in the average time.

The total time for CVS followed the trend of bone wear time (r = 0.89); in T1, because of the direct influence of curettage time (r = 0.94), the mean time for CVS was greater than that in T2, although it was not statistically significant.

**Table 1:** Mean and standard deviations of the variables evaluated in cervical ventral slot (CVS) between C3-C4 in rabbits with piezosurgery (T1) and conventional technique with high-speed burs (T2)*.

Variables	T1	T2
Weight (kg)	3.35 ± 0.32 a	3.29 ± 0.59 a
Access time (min)	9 min 24 s ± 3 min 0 s a	8 min 2 s ± 2 min 35 s a
Time of use of the appliance (min)	8 min 43 s ± 2 min 18 s a	7 min 35 s ± 3 min 20 s a
Curettage time (min)	6 min 8 s ± 3 min 27 s a	4 min 0 s ± 1 min 39 s b
CVS time (min)	14 min 51 s ± 5 min 18 s a	11 min 35 s ± 3 min 46 s a
Total time (min)	24 min 34 s ± 5 min 31 s a	19 min 37 s ± 5 min 30 s b
Room temperature (ºC)	24.57 ± 1.84 a	25.11 ± 1.75 a
Temperature of the irrigation solution (ºC)	23.70 ± 1.52 a	24.23 ± 1.87 a
Temperature before CVS (ºC)	31.85 ± 1.73 a	30.81 ± 2.53 a
Temperature during CVS (ºC)	27.59 ± 2.26 a	27.39 ± 2.00 a
Temperature after CVS (ºC)	26.59 ± 2.08 a	26.56 ± 2.39 a
Difference between the temperatures before and after the CVS (ºC)	5.25 ± 1.09 a	4.25 ± 1.20 b

CVS time: time of use of the apparatus added to the curettage time;

* different letters show differences between treatments (p < 0.05) by the Student’s t-test.

As the total time was directly related to the time of surgical access and CVS, it was significantly higher in T1 than in T2. Therefore, the use of piezoelectric apparatus resulted in longer surgical procedures than that when the conventional CVS technique was used.

### Temperatures

The temperatures of the surgery room and the irrigation solution were not significantly different between the groups since they were adequately controlled to maintain them at approximately 25 ºC.

Regarding the temperatures at the site of the osteotomy before, during, and after CVS, it was observed that the temperatures in piezosurgery were slightly higher than those with the conventional technique, especially before CVS, but without statistical significance. Therefore, when the difference between the initial and final temperatures was calculated, the final result was statistically significantly higher in T1 than that in T2.

### Visibility of the surgical field

According to Mann-Whitney test, the visibility of the surgical field was significantly (p < 0.01) better in T1, in which discrete bone bleeding was observed in only one animal. In T2, discrete bone bleeding and foam formation were observed in four animals, and moderate bone bleeding was seen in two animals and required a hemostatic sponge.

### Intraoperative complications

The only intraoperative complication in both groups was vertebral venous sinus hemorrhage during the curettage stage. According to Mann-Whitney test, it was more frequent in T1 than in T2 (p < 0.01). Hemorrhage was observed in four cases in T1 and two cases in T2.

### Postoperative neurological evaluation

Transient proprioceptive deficits in the postoperative period were observed in three animals (7, 9 and 15) in T1, with recovery within 72 hours of the surgery. None of the animals in T2 developed postoperative neurological changes.

### Anesthetic monitoring

The parameters related to anesthesia monitoring before, during, and after CVS are presented in Table 2. There were no significant differences (p < 0.05) between the groups in any of these parameters (Student’s t-test).

**Table 2:** Mean and standard deviation of anesthesia monitoring data before, during and after cervical ventral slot (CVS) between C3-C4 in rabbits with piezosurgery (T1) and conventional technique with high-speed burs (T2).

	Treatment	Before CVS	During CVS	After CVS
Heart rate (bpm)	T1	198.83 ± 19.17	184.77 ± 21.12	185.16 ± 17.40
	T2	196.12 ± 17.27	193.86 ± 15.45	185.98 ± 24.31
Respiratory rate (mpm)	T1	25.21 ± 5.24	25.20 ± 7.21	24.35 ± 7.80
	T2	23.36 ± 6.64	25.47 ± 4.08	25.70 ± 4.35
Systolic arterial pressure (mmHg)	T1	105.87 ± 9.63	102.76 ± 11.47	106.15 ± 9.97
	T2	104.81 ± 10.92	101.28 ± 11.66	102.54 ± 6.89
Diastolic arterial pressure (mmHg)	T1	47.82 ± 11.99	45.28 ± 11.74	45.97 ± 10.68
	T2	45.72 ± 9.35	43.78 ± 7.70	43.93 ± 8.92
Mean arterial pressure (mmHg)	T1	74.16 ± 7.52	71.82 ± 6.86	72.28 ± 5.14
	T2	73.57 ± 8.45	70.59 ± 9.10	70.77 ± 7.13
Oxygen saturation (%)	T1	98.71 ± 0.41	98.66 ± 0.46	98.59 ± 0.57
	T2	98.73 ± 0.50	98.84 ± 0.30	98.52 ± 0.67
Concentration of carbon dioxide at the end of expiration (mmHg)	T1	47.08 ± 6.95	45.91 ± 4.06	45.69 ± 5.59
	T2	48.04 ± 4.87	47.08 ± 6.24	47.70 ± 4.27
Body temperature (ºC)	T1	38.90 ± 0.67	38.68 ± 0.70	38.38 ± 0.75
	T2	38.72 ± 0.59	38.39 ± 0.75	38.08 ± 0.80
Isoflurane (%)	T1	1.85 ± 0.43	1.60 ± 0.50	1.40 ± 0.53
	T2	1.79 ± 0.39	1.57 ± 0.36	1.31 ± 0.24
Fluid therapy (mL/h)	T1	33.68 ± 3.24	41.91 ± 31.62	33.68 ± 3.24
	T2	34.68 ± 5.73	40.04 ± 23.53	35.27 ± 7.15

### Histopathological analysis

#### Group A (T1): 14 postoperative day

**Figure 1:**
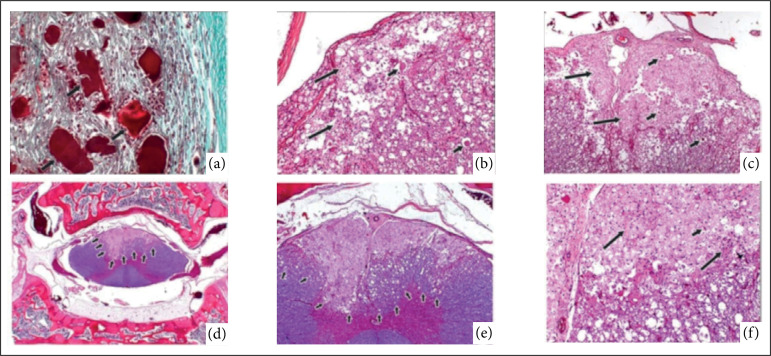
Photomicrographs of the histopathological study of rabbits (*Oryctolagus cuniculus*) submitted cervical ventral slot (CVS) between C3-C4 with piezosurgery (T1) and conventional technique with high-speed burs (T2). **(a)** Histological section of the surgery site (animal 19, T1, Group A): note the degenerated muscle fibers (*arrows*) and surrounded by mononuclear inflammatory cells (Masson’s trichrome, x40); **(b)** Histological section of the spinal cord (animal 7, T1, Group A): note the axonal vacuolization, spherocytes (*short arrows*) and *Gitter cells* (hematoxylin and eosin, x20); **(c)** Histological section of the spinal cord (animal 13, T1, Group A): note the axonal vacuolization, spherocytes and *Gitter cells* (hematoxylin and eosin, x10); **(d)** Histological section of the spinal cord (animal 9, T1, Group C): note the lesion area (*arrows*) (hematoxylin and eosin, x1); **(e)** Previous figure in the highest magnification (x5); **(f)** Previous figure in higher magnification (x20): note the axonal vacuolization, spherocytes and *Gitter cells* (Sampling date: September 29, 2017).

On the 14^th^ postoperative day, vascularized connective tissue and moderate inflammatory infiltrate of the chronic active type were observed at the site of the lesions. The bone window was still open, but filled with granulation tissue, and there was a great deal of bone debris enveloped by the inflammatory reaction. The muscle tissue near the CVS site was degenerate and had syncytium (Fig. 1a). At the edges of the lesions, the spongy bone was hyperplastic. The impairment of the spinal cord ranged from mild to moderate (Fig. 1 b-c, Table 3).

**Table 3:** Histopathological changes classified according to the type and degree of lesion present in the spinal cord of rabbits after cervical ventral slot (CVS) with piezosurgery (T1) and conventional technique with high-speed burs (T2)*,#.

Treatment	Experimental group	Animal	Type of injury	Degree
T1	A^A^	1#	Discrete Wallerian degeneration	+
		7#,$	Malacia with spherocytes and *Gitter cells*	++
		13	Malacia with spherocytes and *Gitter cells*	++
		19	Absent	-
		25	Malacia with spherocytes and *Gitter cells*	++
T2	B^B^	2	Absent	-
		8	Discrete Wallerian degeneration	+
		14	Discrete Wallerian degeneration	+
		20	Absent	-
		26#	Discrete Wallerian degeneration	+
T1	C^A^	3	Technical artifact**	
		9$	Malacia with spherocytes and *Gitter cells*	++
		15$	Malacia with spherocytes and *Gitter cells*	++
		21	Malacia with spherocytes and *Gitter cells*	++
		27	Absent	-
T2	D^B^	4	Absent	-
		10	Absent	-
		16	Absent	-
		22	Absent	-
		28	Absent	-
T1	E^A^	5	Absent	-
		11#	Absent	-
		17#	Absent	-
		23	Wallerian degeneration with spherocytes	+
		29	Discrete Wallerian degeneration	+
T2	F^B^	6	Absent	-
		12	Absent	-
		18	Absent	-
		24	Absent	-
		30#	Technical artifact**	

A and B A and B: euthanasia at 14 postoperative days;

C and D: euthanasia at 28 postoperative days; E and F: euthanasia at 56 postoperative days; -: absent lesions; +: mild lesions; ++: moderate lesions; +++: severe lesions;

* different letters show difference (p < 0.05) by the Mann-Whitney test, in which capital letters represent differences between treatments (T1 and T2) for the experimental groups with the same postoperative period;

# animals that presented intraoperative venous sinus hemorrhage;

$ animals that presented proprioceptive deficits in the postoperative period;

** artifact technique indicates a damaged sample during processing, making it impossible to read;

#different letters show difference (p < 0.05) by the Mann-Whitney test, in which capital letters represent differences between treatments (T1 and T2) for the experimental groups with the same postoperative period.

#### Group B (T2): 14 postoperative days

There were no signs of inflammation in the cicatricial focus in 3/5 animals (8, 20 and 26), and the signs were discrete in the other two (2 and 14)—consisting mainly of small amounts of monocytes, macrophages, lymphocytes, and plasma cells. Skeletal muscle degeneration was mainly represented by the formation of syncytium and edematous segmented muscle fibers and was detected in 4/5 animals (2, 8, 14 and 20). Additionally, mild-to-moderate hemorrhage at the CVS site was present in 4/5 animals (2, 8, 14 and 20). With respect to the spinal cord, there were no morphological alterations in 2/5 (2 and 20) animals, while in the other three (8, 14 and 26) the morphological changes were mild (Table 3).

#### Group C (T1): 28 postoperative days

On the 28^th^ postoperative day, at the site of the lesion, vascularized connective tissue was mature with occasional collagenization and sparse inflammatory cells composed mainly of mononuclear cells. Adjacent to the scars, the muscle fibers were enveloped by connective tissue in an isolated manner and, underlying the scar, there was a bony callus filling the window, in which it varied from discrete to exuberant (Fig. 1 d-f).Regarding the analysis of the spinal cord, only one sample was without morphological alterations, and the others were classified as moderate (Table 3).

#### Group D (T2): 28 postoperative days

**Figure 2:**
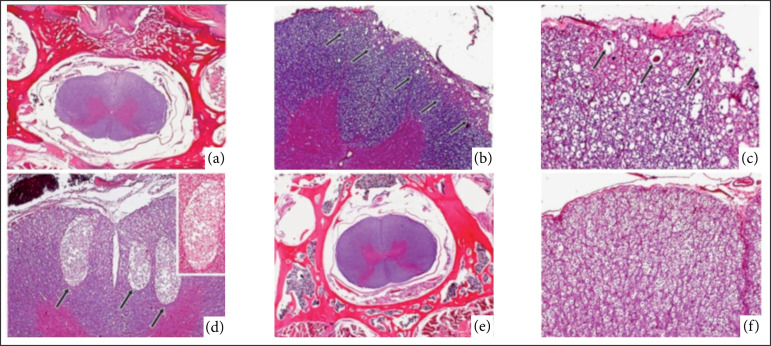
Photomicrographs of the histopathological study of rabbits (*Oryctolagus cuniculus*) submitted to cervical ventral slot (CVS) between C3-C4 with piezosurgery (T1) and conventional technique with high-speed burs (T2). **(a)** Histological section of the spinal cord (animal 28, T2, group D), with no morphological changes worthy of note (hematoxylin and eosin, x1); **(b)** Histological section of the spinal cord (animal 29, T1, group E): note the malacia area at the top of the image (*arrows*) (hematoxylin and eosin, x5); **(c)** Previous figure in higher magnification (x20): note the vacuolization and the spherocytes (*arrows*); **(d)** Histological section of the spinal cord (animal 23, T1, group F): note the areas of malacia (*arrows*) (hematoxylin and eosin, x5). Right upper corner: figure in highest magnification (x10); **(e)** Histological section of the spinal cord (animal 24, T2, group F): with no morphological changes worthy of note (hematoxylin and eosin, x1);**(f)** Previous figure in highest magnification (x10) (Sampling date: September 29, 2017).

In this group, the same pattern of soft tissue healing as in group C was observed along with discrete amounts offoreign body-type giant cells in the superficial regions of the scars. The histological findings of the bony callus were also similar to those in group C. The notoriety of this group was due to the morphological characteristics of the nervous tissue, with total preservation of histology in all five samples (Fig. 2a, Table 3).

#### Group E (T1): 56 postoperative days

In this group, a scar was observed from the subcutaneous tissue to the bone callus. The bone callus varied from discrete to exuberant, with cartilaginous metaplasia and the presence of fibrocartilage in a random and inconstant manner in the samples. The newly formed bone was spongy, and the gaps had viable hematopoietic cells. There were no histopathological changes in the spinal cord in 3/5 animals (5, 11 and 17). However, in two animals (23 and 29), such changes were graded as mild (Fig. 2 b-d, Table 3).

#### Group F (T2): 56 postoperative days

In this group, the morphological pattern of healing and bone callus were similar to the findings in group E.The morphological characteristics of the spinal cord remained unchanged in 4/4 animals (6, 12, 18 and 24) (Fig. 2 e-f, Table 3).

Therefore, in the present study, a greater number of histopathological spinal cord lesions was observed with piezosurgery—especially at 14 and 28 POD—, that were characterized by malacia, spherocytes, and *Gitter cells*. In contrast, with the conventional technique, only mild Wallerian degeneration was observed 14 days after the surgery.

## Discussion

The piezosurgery allows easy intraoperative operation of its instruments and accurate bone cutting because of the use of ultrasonic microvibrations to perform osteotomies. In contrast, the conventional instruments, such as bone drills and oscillatory saws, work with macrovibrations, which make precise cutting impossible due to the loss of tactile skill and sensitivity of the surgeon^12^-^14^. Such differences agree with the findings of this study, which revealed precise and regular osteotomy with the piezoelectric apparatus and irregular elliptical cuts with the conventional technique.

The present study also showed that the total time of piezosurgery was longer than that of the conventional technique. According to Hennet^5^ and Barone *et al.* 15, piezosurgery usually requires longer surgical time due to the smaller size of its cuts when compared with traditional instruments. Therefore, depending on the structure and bone thickness involved, the duration of the procedure can increase by up to five times^16^. However, many studies have presented varying results when comparing the surgical times of the two techniques^11^,^15^-^19^. This variation may be related to the type of surgery, structures involved, and instruments used^5^.

The curettage stage contributed significantly to the higher total piezosurgery time. This was due, in part, to the complete preservation of the intervertebral disc at theend of the corpectomy in T1 group, possibly due to the absence of signs of calcification of this material and deprivation of cut of the non-mineralized tissues by the piezoelectric apparatus. Therefore, it was necessary touse the curette to perform the discectomy, and this excessive manipulation increased the risk of injury to the spinal cord and vertebral venous sinus, as demonstrated by four cases of hemorrhage. Because of such complications in the first few procedures, there was an increase in the surgical time inthe others due to greater care being taken for curettage. In contrast, high-speed burs remove any type of tissue and, therefore, during the initial bone removal they already remove most of the intervertebral disc together with the vertebral bone, reducing the amount of material needed to be cured and, consequently, reducing manipulation.

The rotary cutting instruments produce excessively high temperatures during osteotomy^20^,^22^, whereas the piezoelectric device—since it does not require pressure against the bone to make the cut—presents less chances of thermal injury^5^. Nevertheless, cooling with saline solution is recommended during both techniques. In a study by Kerawala *et al.* 22, irrigation had the greatest effect in reducing the final temperature at the site of bone perforation; therefore, its absence resulted in surgical site temperatures above 70 ºC. In the *in vivo* experiments described here, the final CVS site temperatures in both techniques were below the critical levels for intraosseous hyperthermia (40–41 ºC) and bone necrosis (47 ºC)^23^. Therefore, it can be stated that the use of the irrigation solution at a temperature of 25 ºC and rate of 30 mL/minwas effective in reducing the heat produced in both techniques.

The superior surgical visibility in piezosurgery was described in the present study and reported in several other studies that made use of piezoelectric devices^5^,^6^,^9^,^11^,^12^,^24^. This is a consequence of the interaction between the ultrasonic vibrations and the intraoperative irrigation jet, which reduces the hemorrhage within the surgical field, removes the bony debris from the cutting surface, and provides cooling^6^. Additionally, the better visibility combined with the sparing of soft tissues by the piezoelectric apparatus resulted in minimal damage to the blood vessels^5^. According to Landes *et al.* 13, bleeding can be reduced by up to 25%–30% when compared with the conventional techniques.

Regarding the histopathological evaluation of the spinal cord of the two proposed treatments, a greater number of lesions were observed with piezosurgery, especially at 14 and 28 POD. It is believed that the alterations observed in the ventral tracts of the spinal cord may be because of iatrogenic trauma caused by excessive manipulation of the curette within the vertebral canal during discectomy and the piezoelectric tip, which—in order to produce single block cuts—enters the spinal canal blindly. Therefore, the planned surgical access and determination of the thickness of the vertebral body from imaging allow the use of tips with millimeter markings, in order to guide the limit of its introduction into the vertebral body and avoid iatrogenic damages to the spinal cord. Alternatively, a first tip for *cis-*cortical osteotomy can be used and, then, a smaller and more delicate second tip can be used for subsequent excision of the *trans-*cortical osteotomy. Therefore, studies regarding piezoelectric surgery emphasize the importance of having several tips available, with different angulations and diameters, for more accurate bone cuts^16^,^25^,^26^.

Schaeren *et al.* 27 carried out a study to assess the potential damage of piezoelectric instruments to a peripheral nerve upon direct contact under different forces, and this contact with the nerve was also applied without ultrasonic activation. They conclude that the direct exposure of peripheral nerve to piezoelectric device did not dissect the nerve, but induced structural and functional damage. The frequency and extent of functional damage were higher with increased pressure applied on the nerve, but not by activation of ultrasonic vibration. Therefore, it is believed that this is what happened to the animals in T1 group. The mechanical contact of the tip of the piezoelectric instrument with the spinal cord, andnot the mechanism of action of the device, led to reversible structural and functional damage.

In the conventional technique with high-speed burs, great care is taken to avoid the entry of the drill into thevertebral canal, as it is known that its contact withthe spinal cord will cause irreversible damage to the neural tissue. Thus, the drill is used only until the internal cortex, and then special instruments are used to complete the spondylectomy and enter the vertebral canal. Therefore, according to our results, the lesser spinal cord damage in T2 group is related to the cautious surgical technique used with the conventional device and not to the safety of the device itself when having direct contact with the nervous tissue.

Therefore, as long as there is no mechanical contact between the tip of the piezoelectric instrument and the spinal cord, it is possible to use this device in neurosurgical procedures safely and promoting precise and *en bloc* bone cutting, excellent intraoperative visibility and greater dexterity in handling the instrument. Thus, the limitations involve the cost of acquiring the device, the need for preoperative planning to opt for the ideal size tip according to the patient’s size and, consequently, the need to purchase different tips.

## Conclusions

CVS in rabbits with piezosurgery promoted precise bone cutting, excellent visibility of the operative field and ease of handling of the instrument. However, when compared with the conventional technique, it required a longer total surgical time and resulted in a greater number of intraoperative complications—such as microscopic lesions in the spinal cord—and transient neurological deficits in the postoperative period. It is believed that these injuries were triggered by the mechanical touch of the tip of the piezoelectric instrument in the spinal cord and not by the mechanism of action of the device. Therefore, performing the proper surgical planning and having different tips available, piezosurgery is applicable in neurosurgical procedures.
